# A population‐based study on the prognostic impact of primary tumor sidedness in patients with peritoneal metastases from colon cancer

**DOI:** 10.1002/cam4.3243

**Published:** 2020-07-02

**Authors:** Nadine L. de Boer, Koen Rovers, Jacobus W.A. Burger, Eva V. E. Madsen, Alexandra R. M. Brandt‐Kerkhof, Niels F. M. Kok, Johannes H. W. de Wilt, Philip H. de Reuver, Amanda Bos, Ignace H. J. T. de Hingh, Cornelis Verhoef

**Affiliations:** ^1^ Department of Surgical Oncology Erasmus MC Cancer Institute Rotterdam the Netherlands; ^2^ Department of Surgery Catharina Cancer Institute Eindhoven the Netherlands; ^3^ Department of Surgery Antoni van Leeuwenhoek Hospital The Netherlands Cancer Institute Amsterdam the Netherlands; ^4^ Department of Surgical Oncology Radboud UMC Nijmegen the Netherlands; ^5^ Department of Research & Development Netherlands Comprehensive Cancer Organisation (IKNL) Utrecht the Netherlands

**Keywords:** Colon cancer, peritoneal metastases, primary tumor sidedness

## Abstract

Primary tumor location is an established prognostic factor in patients with (metastatic) colon cancer. Colon tumors can be divided into left‐sided and right‐sided tumors. The aim of this study was to determine the impact of primary tumor location on treatment and overall survival (OS) in patients with peritoneal metastases (PM) from colon cancer. This study is a retrospective, population‐based cohort study. Records of patients diagnosed with colon cancer and synchronous PM, from 1995 through 2016, were retrieved from the Netherlands Cancer Registry (NCR). Data on diagnosis, staging, and treatment were extracted from the medical records by specifically trained NCR personnel. Information on survival status was updated annually using a computerized link with the national civil registry. In total, 7930 patients were included in this study; 4555 (57.4%) had a right‐sided and 3375 (42.6%) had a left‐sided primary tumor. In multivariable analysis right‐sided primary tumor was associated with worse OS (HR: 1.11, 95% CI 1.03‐1.19, *P* = .007). Of all patients diagnosed with PM, 564 (7.1%) underwent cytoreductive surgery and hyperthermic intraperitoneal chemotherapy (CRS‐HIPEC). Patients with left‐sided primary tumors were more often candidates for CRS‐HIPEC (6.5% vs. 8.0%, *P* = .008). OS of patients with right‐ and left‐sided tumors who underwent CRS‐HIPEC did not significantly differ. In conclusion, primary right‐sided colon cancer was an independent prognostic factor for decreased OS in patients diagnosed with synchronous PM. In patients treated with CRS‐HIPEC location of the primary tumor did not influence survival.

## Graphical abstract

Primary tumor location is an established prognostic factor for patients with colon cancer. Little is known about the impact of tumor location on the outcomes of patients with peritoneal metastases. In a population‐based study in the Netherlands, primary right‐sided colon cancer was an independent prognostic factor for decreased survival in patients with synchronous peritoneal metastases. In patients with synchronous peritoneal metastases treated with CRS‐HIPEC, location of the primary tumor did not influence survival.

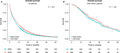

## 1 INTRODUCTION

Approximately 5% of all patients with primary colon cancer, present with synchronous peritoneal metastases (PM).^1^ Cytoreductive surgery and hyperthermic intraperitoneal chemotherapy (CRS‐HIPEC) has led to an increased overall survival (OS) of patients with PM from colon cancer over the last two decades, but also has considerable postoperative morbidity and mortality rates.^2^, ^3^, ^4^, ^5^, ^6^, ^7^, ^8^, ^9^, ^10^

Examples of risk factors for patients with PM associated with impaired survival include the extent of peritoneal disease, extraperitoneal metastases, advanced primary tumor stage, nodal metastases, poor tumor differentiation, and poor performance status.^10^, ^11^, ^12^ Several studies have shown that primary tumor location also is a prognostic factor in patients with (metastatic) colon cancer.^13^, ^14^, ^15^, ^16^ Colon tumors can be divided into left‐sided and right‐sided tumors. Right‐ and left‐sided primary tumors differ in embryological origin (mid‐gut vs hind‐gut), bacterial flora and tumor biology (ie, mutational status and microsatellite instability (MSI)).^17^, ^18^, ^19^ In patients with metastatic colon cancer it is known that patients with right‐sided tumors have worse tumor response rates to treatment with systemic chemotherapy, in both an adjuvant and palliative setting, and impaired survival outcomes. Other studies have shown that in patients who underwent resection for liver metastases, right‐sided primary tumor was independently associated with impaired outcomes as well.^20^, ^21^, ^22^, ^23^, ^24^ However, little is known about the role of primary tumor location in patients with PM, and patients with PM undergoing CRS‐HIPEC.^25^, ^26^ The aim of this study was to assess the impact of the primary tumor location in all patients with PM of colon cancer on a population‐based level.

## 2 METHODS

### 2.1 Collection of data

This was a retrospective, population‐based cohort study in the Netherlands, a high‐income Western‐European country with currently over 17 million inhabitants. Data on patients with colon cancer diagnosed with synchronous PM from 1995 through 2016, were retrieved from the Netherlands Cancer Register (NCR), after formal approval by the NCR Monitoring Committee. The NCR collects data on all patients (no age restrictions) diagnosed with cancer in the Netherlands based on notification of newly diagnosed malignancies by the national automated pathological archive and on hospital discharge diagnoses. Before 2008, data regarding the location of metastases were collected for several regions in the Netherlands, since 2008 these data are collected on a population‐based level. Information on baseline characteristics, staging, treatment, and survival was extracted routinely from the medical records by trained NCR personnel. Information on survival status is updated annually using a computerized link with the Dutch population register. For the present analysis, survival information was updated to 31 January 2018. The NCR does not collect data on disease‐free survival.

### 2.2 Patient selection

All patients diagnosed with colon cancer and synchronous PM between 1995 and 2016 were eligible for inclusion in this study. As all data were anonymized, no formal approval of a medical ethic committee was required under Dutch law. Right‐sided primary tumors were defined as those of the cecum, ascending colon, hepatic flexure, and transverse colon. Left‐sided tumors were defined as those of the splenic flexure, descending colon, sigmoid, or rectosigmoid. Patients with an unknown location of the primary tumor site and a primary appendiceal‐ or rectum tumor were excluded from this study.

### 2.3 Definition and outcome parameters

Treatment approach was divided in treatment with curative intent (patients that underwent CRS‐HIPEC) or treatment with palliative intent (systemic chemotherapy, palliative surgery or best supportive care). All CRS‐HIPEC procedures were performed according to the standardized Dutch CRS‐HIPEC protocol.^7^, ^27^ Main outcome of this study was overall survival (OS). OS was calculated from the date of diagnosis until death or last follow‐up. OS after CRS‐HIPEC was calculated from the date of surgery until death or last follow‐up.

### 2.4 Statistical analysis

Continuous variables were presented as median with interquartile range (IQR). Categorical variables were presented as absolute numbers and percentages. Baseline characteristics were compared using the chi‐squared test (for proportions) and Mann‐Whitney U test (for continuous data). The Kaplan‐Meier method was used for survival analysis and comparisons between groups were made using log rank test. Patients were censored when alive at last follow‐up date. Univariable and multivariable Cox proportional‐hazards models were constructed to identify prognostic factors and hazard ratios (HR) and 95% confidence intervals (CI) for these factors were calculated. Variables with a *P*‐value < .05 or clinically relevant variables were included in the multivariable analysis. Two‐sided *P*‐values < .05 were considered statistically significant. Statistical analyses were performed using Statistical Package for Social Sciences (SPSS), Version 24.0.0 for Windows (IBM Corporation).

## 3 RESULTS

### 3.1 Patient characteristics

#### 3.1.1 All patients diagnosed with peritoneal metastases

A total of 9759 patients were diagnosed with synchronous PM during the inclusion period. A total of 1839 patients (18.7%) were excluded because of unknown primary tumor site (n = 529, 5.4%), primary appendiceal tumor (n = 377, 3.9%), or primary rectal cancer (n = 923, 9.5%). Therefore, 7930 patients remained in the study for analysis. Baseline characteristics of the right‐ and left‐sided tumor group are described in Table 1.

**TABLE 1:** Baseline characteristics of all patients diagnosed with peritoneal metastasis

Baseline variables	Right‐sided N = 4555 (57.4%) Median [IQR]/N(%)	Left‐sided N = 3375 (42.6%) Median [IQR]/N (%)	*P* value
Age at diagnosis, years	70 [62‐78]	69 [60‐77]	<.001
Sex			<.001
Male	2102 (46.1)	1833 (54.3)	
Female	2453 (53.9)	1542 (45.7)	
Year of diagnosis			.035
1995‐1999	410 (55.9)	323 (44.1)	
2000‐2004	530 (53.9)	453 (46.1)	
2005‐2009	1293 (57.1)	970 (42.9)	
≥2010 (2016)	2322 (58.8)	1629 (41.2)	
Location primary			N/A
Cecum	2161 (47.4)		
Ascending colon	1186 (26.0)		
Hepatic flexure	487 (10.7)		
Transverse colon	721 (15.8)		
Splenic flexure		377 (11.2)	
Descending colon		353 (10.5)	
Sigmoid colon		2301(68.2)	
Recto sigmoid		344 (10.2)	
pT‐stage primary			.225
T1‐2	31 (0.7)	30 (0.9)	
T3‐4	2364 (51.9)	1675 (49.6)	
*Unknown*	*2160 (47.4)*	*1670 (49.5)*	
pN‐stage primary			<.001
N0	268 (5.9)	261 (7.7)	
N+	2039 (44.8)	1358 (40.2)	
*Unknown*	*2248 (49.4)*	*1756 (52.0)*	
Extraperitoneal metastasis			<.001
Yes	2130 (46.8)	1737 (51.5)	
No	2425 (53.2)	1638 (48.5)	
Mucinous histology			.894
Yes	923 (20.3)	688 (20.4)	
No	3632 (79.7)	2687 (79.6)	
Differentiation			<.001
Well	112 (2.5)	106 (3.1)	
Moderate	1448 (31.8)	1262 (37.4)	
Poor	1276 (30.0)	730 (21.6)	
*Unknown*	*1719(37.7)*	*1277(37.8)*	
CTx^b^			.082
Yes	2028 (44.5)	1569 (46.5)	
No	2527 (55.5)	1806 (53.5)	
CRS‐HIPEC			.008
Yes	294 (6.5)	*270 (8.0)*	
No	4255 (93.4)	*3103 (91.9)*	
*Unknown*	*6 (0.1)*	*2 (0.1)*	

^a^ *CTx* chemotherapy, *CRS‐HIPEC* cytoreductive surgery and hyperthermic intraperitoneal chemotherapy.

^b^ CTx either administered in the palliative setting or in neoadjuvant or adjuvant setting or both.

Patients with PM from right‐sided primary tumors were older and more frequently females when compared to the left‐sided tumor group. The right‐sided tumor group also had more node positives and poor tumor differentiation was more common. In the left‐sided tumor group extraperitoneal metastases were more frequent. Patients with PM from left‐sided tumors underwent CRS‐HIPEC more frequently (all factors *P* < .01).

#### 3.1.2 Patients treated with CRS‐HIPEC

Of all 7930 patients included in this study, 564 patients (7.1%) were treated CRS‐HIPEC (Table 2). Female sex was more common in the right‐sided tumor group. No other significant differences were observed at baseline.

**TABLE 2:** Baseline characteristics of patients treated with CRS‐HIPEC

Baseline variables	Right‐sided N = 294 (52.1%) Median [IQR]/N (%)	Left‐sided N = 270 (47.9%) Median[IQR]/N (%)	*P* value
Age at diagnosis	62 [54‐67]	61 [55‐68]	.880
Sex			
Male	123 (41.8)	146 (54.1)	.004
Female	171 (58.2)	124 (45.9)	
Year of diagnosis			
1995‐2009	48 (44.9)	59 (55.1)	.095
2010‐2016	246 (53.8)	211 (46.2)	
Location primary			N/A
Cecum	155 (52.7)	—	
Ascending colon	68 (23.1)	—	
Hepatic flexure	18 (6.1)	—	
Transverse colon	53 (18)	—	
Splenic flexure	—	22 (8.1)	
Descending colon	—	27 (10.0)	
Sigmoid colon	—	205 (75.9)	
Recto sigmoid	—	16 (5.9)	
pT‐stage primary			.201
T1‐2	6 (2.0)	2 (0.7)	
T3‐4	279 (94.9)	255 (94.4)	
*Unknown*	*9 (3.1)*	*13 (4.9)*	
pN‐stage primary			.746
N0	46 (15.6)	44 (16.3)	
N+	240 (81.6)	213 (78.9)	
*Unknown*	*8 (2.7)*	*13 (4.8)*	
Extraperitoneal metastasis			
Yes	*36 (12.2)*	*42 (15.6)*	*.255*
*No*	*258 (87.8)*	*228 (84.4)*	
Mucinous histology			.288
Yes	105 (35.7)	85 (31.5)	
No	189 (64.3)	185 (68.5)	
Differentiation			.652
Well	8 (2.7)	6 (2.2)	
Moderate	139 (47.3)	140 (51.9)	
Poor	63 (21.4)	53 (19.6)	
*Unknown*	*84 (28.6)*	*71 (26.3)*	
Perioperative CTx^b^			.736
Yes	201 (68.4)	181 (67.0)	
No	93 (31.6)	89 (33.0)	
Prior Surgery of primary			.059
Yes	153 (52.0)	117(43.4)	
No	124 (42.2)	132 (48.9)	
*Unknown*	*17 (5.8)*	*21 (7.8)*	

^a^ *CTx* chemotherapy, *CRS‐HIPEC* cytoreductive surgery and hyperthermic intraperitoneal chemotherapy.

^b^ CTx either administered in the neoadjuvant setting or adjuvant setting or both.

#### 3.1.3 Patients receiving palliative treatment

Most patients (92.9%) were treated with palliative intent. Table S1 includes baseline characteristics of these patients.

### 3.2 Overall survival

#### 3.2.1 Overall survival of all patients diagnosed with peritoneal metastases

**FIGURE 1:**
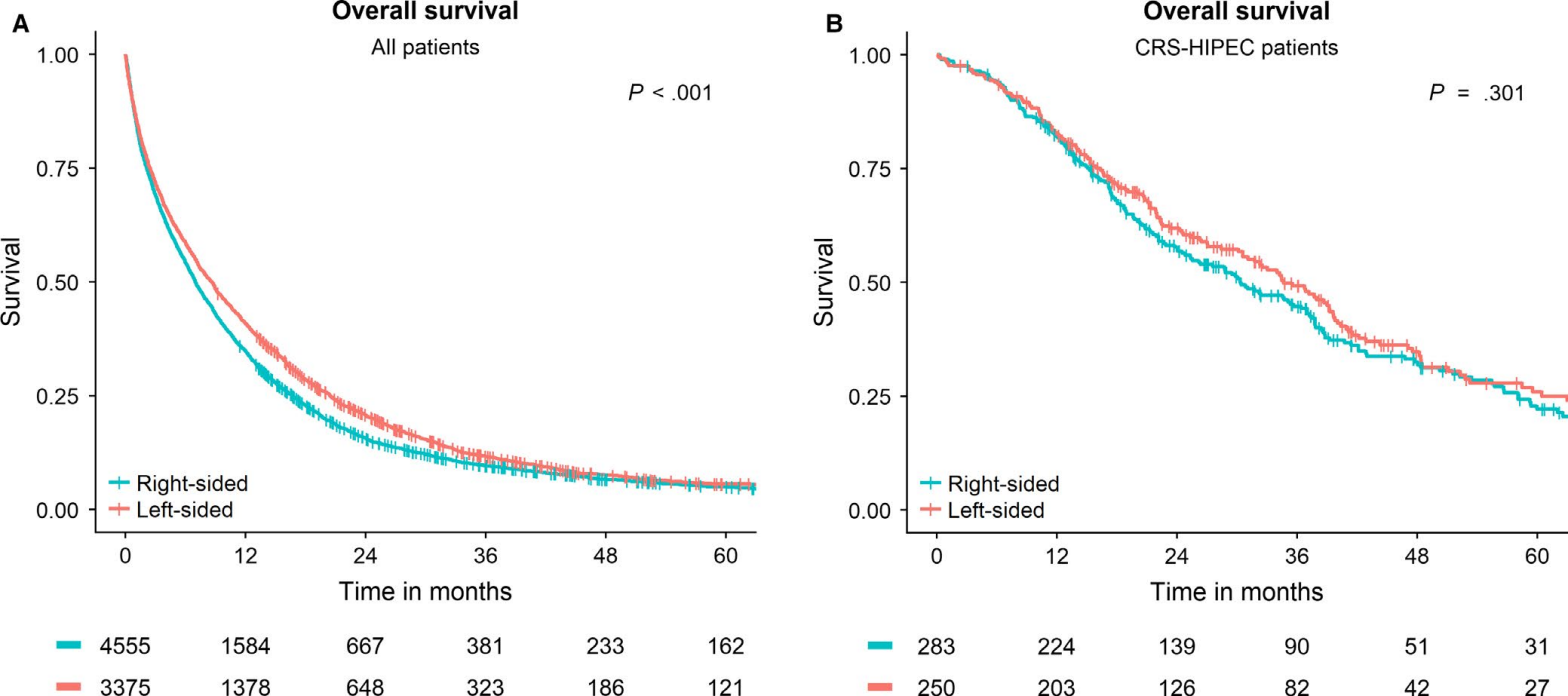
Overall Survival curves of patients with synchronous peritoneal metastases from colon cancer. A, Overall survival of all patients after diagnosis. B, Overall survival of patients after undergoing CRS‐HIPEC

Median follow‐up of all patients was 7.5 months [IQR 2.2‐17.6]. Of the 7959 patients included, 7439 (93.8%) died during the course of the study. The survival curve of these patients is shown in Figure 1A. Median OS of all patients diagnosed with synchronous PM was 7.5 months [IQR 2.2‐18.2]. Median OS of right‐sided tumors was worse as compared to the left‐sided tumors (OS 7.0 months, IQR 2.1‐16.7 vs OS 8.6 months, IQR 2.4‐20.4, *P* < .001).

After correction for possible confounders, multivariable analysis showed that a right‐sided primary tumor was an independent prognostic factor for impaired survival (HR 1.11, 95% CI 1.03‐1.19, *P* = .007). Other prognostic factors associated with worse survival outcomes were age at diagnosis, advanced tumor stage of the primary tumor, nodal metastasis, existence of extraperitoneal metastasis, and poor tumor differentiation. Patients diagnosed within or after 2010, patients that received chemotherapy and patients that underwent CRS‐HIPEC had significant better outcomes (all factors *P* < .01). All results of univariable and multivariable analysis are shown in Table 3.

**TABLE 3:** Univariable and Multivariable Cox regression analysis for OS of all patients after diagnosis with peritoneal metastasis

Factor	Univariable analysis			Multivariable analysis		
	HR	95% CI	*P* value	HR	95% CI	*P* value
Age at diagnosis (cont.)	1.03	1.02‐1.03	<.001	1.01	1.01‐1.02	<.001
Female sex	1.05	1.00‐1.09	.057			
Year of incidence ≥ 2010	0.87	0.83‐0.91	<.001	0.89	0.82‐0.96	.002
Right‐sided primary	1.12	1.07‐1.18	<.001	1.11	1.03‐1.19	.007
pT3‐T4 (primary)	1.85	1.40‐2.46	<.001	2.10	1.47‐3.00	<.001
pN + CRC	1.67	1.51‐1.84	<.001	1.83	1.63‐2.05	<.001
Extraperitoneal metastasis	1.41	1.34‐1.47	<.001	1.80	1.66‐1.95	<.001
Poor differentiation	1.53	1.44‐1.62	<.001	1.43	1.33‐1.55	<.001
Mucinous histology	0.80	0.76‐0.85	<.001	1.01	0.93‐1.11	.771
Chemotherapy	0.46	0.44‐0.48	<.001	0.48	0.44‐0.52	<.001
CRS‐HIPEC	0.29	0.26‐0.32	<.001	0.57	0.50‐0.65	<.001

Abbreviations: CI, confidence interval; CRC, colorectal cancer; CRS‐HIPEC, cytoreductive surgery and hyperthermic intraperitoneal chemotherapy; HR, hazard ratio; pN+, lymph node positives.

#### 3.2.2 Overall survival of patients treated with CRS‐HIPEC

Median follow up after CRS‐HIPEC was CRS‐HIPEC was 23.7 months [13.6‐39.6]. At last follow up 197 (34.9%) of the patients that underwent CRS‐HIPEC were still alive. OS of patients who underwent CRS‐HIPEC was 33.0 months [IQR 15.5‐59.3]. There was no difference in OS between patients with right‐ or left‐sided primary tumors who underwent CRS‐HIPEC (OS 30.3 months [IQR 15.2‐58.1] vs OS 34.6 months [IQR 16.3‐60.4], *P* = .301). Figure 1B shows the OS of all patients that were treated with CRS‐HIPEC.

The results of univariable and multivariable analysis in patients treated with CRS‐HIPEC are shown in Table 4. In patients who underwent CRS‐HIPEC right‐sided primary tumor was not independently associated with impaired survival. Factors associated that were associated with an impaired OS after CRS‐HIPEC were poor tumor differentiation and nodal metastasis. Perioperative chemotherapy was associated with improved OS after CRS‐HIPEC (all factors *P* < .01).

**TABLE 4:** Univariable and Multivariable Cox regression analysis for OS of patients treated with CRS‐HIPEC

Factor	Univariable analysis			Multivariable analysis		
	HR	95% CI	*P* value	HR	95% CI	*P* value
Age at surgery (cont.)	1.01	1.00‐1.02	.149			
Female sex	0.88	0.71‐1.09	.240			
Year of incidence (≥2010)	1.10	0.84‐1.45	.488			
Right‐sided primary	1.12	0.90‐1.39	.302	1.24	0.96‐1.60	.098
pT3‐T4 (primary)	6.00	0.84‐42.77	.074			
pN + CRC	1.66	1.21‐2.27	.002	1.71	1.16‐2.50	.006
Extraperitoneal metastasis	1.31	0.97‐1.78	.078			
Poor differentiation	1.74	1.34‐2.26	<.001	1.58	1.21‐2.08	.001
Mucinous histology	0.83	0.66‐1.04	.098			
Peri‐operative chemotherapy	0.71	0.57‐0.90	.004	0.65	0.49‐0.86	.003
Prior surgery of primary tumor	0.76	0.61‐0.94	.013	0.82	0.63‐1.06	.127

Abbreviations: CI, confidence interval; CRC, colorectal cancer; CRS‐HIPEC, cytoreductive surgery and hyperthermic intraperitoneal chemotherapy; HR, hazard ratio; pN+, lymph node positives.

#### 3.2.3 Overall survival of patients receiving palliative treatment

Figures S1A‐C show the survival curves of patients treated with palliative intent. In supplementary Table 2, the univariable an multivariable analysis of patients treated in a palliative setting can be found.

## 4 DISCUSSION

This current study showed that patients with synchronous PM from right‐sided colon cancer had a worse prognosis as compared to patients with PM from left‐sided tumors. Patients in the latter group were more frequently candidates for CRS‐HIPEC. Other prognostic factors included age, advanced primary tumor stage (T3‐T4), nodal metastases, poor tumor differentiation, extraperitoneal metastases, and whether or not the patient was treated with systemic chemotherapy and/or CRS‐HIPEC. In patients who underwent CRS‐HIPEC the primary tumor location did not influence survival anymore. Prognostic factors for survival after CRS‐HIPEC did include nodal metastases, poor tumor differentiation, and treatment with systemic perioperative chemotherapy, either administered in the neoadjuvant or adjuvant setting or both.

Prior research has already shown that right‐ and left‐sided colon tumors have different biological behavior and survival outcomes. This study confirms this for patients with synchronous PM. However, in this study primary tumor location did not influence the results after CRS‐HIPEC. This is remarkable since most studies have shown that in patients who underwent resection for other colon cancer metastases (such as liver metastases), right‐sided primary tumor was independently associated with impaired outcomes.^23^, ^28^, ^29^ A possible explanation is that the prognosis of patients with PM is poor in general, and therefore no significant difference was found. Also, the lack of difference in survival outcomes could be due to the relative small sample size of the CRS‐HIPEC group.

Two other cohort studies where recently published regarding the impact of primary tumor location in patients treated with CRS and/or CRS‐HIPEC.^25^, ^26^ In contrast with the current results, a significant impaired DFS and OS was observed for patients with right‐sided tumors after CRS and CRS‐HIPEC. This difference could be due to the fact that both studies excluded all patients with a primary tumor in the transverse colon in their analysis. Others have shown a gradual change, for example, in microenvironment, from the ascending colon to the rectum.^18^, ^30^, ^31^ Excluding all primary tumors from the transverse colon could have resulted in an increased, and significant difference between left and right‐sided tumors. When we excluded all patients with a primary tumor in the transverse colon from our own analysis the difference in OS between patients with right‐ or left‐sided primary tumors who underwent CRS‐HIPEC increased with 1.5 months, to a total difference of 5.8 months (OS 28.8 months [IQR 13.55‐56.7] vs OS 34.6 months [IQR 15.5‐60.4], *P* = .149 (data not shown)). Another possible explanation for the dissimilarity in results, is referral bias. Both studies included only high‐volume referral centers, while we performed a population‐based study. It is published that patients treated in referral centers have different characteristics when compared with the general population.^32^

The results of this study suggest that perioperative chemotherapy results in better OS after CRS‐HIPEC. Possible explanations include a true positive effect of perioperative chemotherapy on patients with synchronous PM, or selection bias. Patients who received treatment with (neo)adjuvant systemic were probably in a better overall condition and did not show progression under neo adjuvant treatment. Patients who received adjuvant chemotherapy are less likely to have experienced very early progression postoperatively or a delayed postoperative course due to severe surgical complications. Therefore this is a selected group. Upfront CRS‐HIPEC is currently standard of care for patients with resectable PM in the Netherlands. However, in other countries perioperative chemotherapy combined with CRS‐HIPEC is common practice. The effect of neoadjuvant systemic therapy for patients with resectable PM is being studied in a national multicenter randomized controlled trial in the Netherlands (CAIRO6 study). In this trial patients are randomized to either upfront CRS‐HIPEC or CRS‐HIPEC plus perioperative systemic chemotherapy. This study will provide the OS data to determine which treatment is superior.^33^

Unfortunately, the majority of patients (>90%) with PM from colon cancer are not eligible for treatment with curative intent. This study shows that patients with PM from right‐sided colon cancer who are treated in a palliative setting have a worse OS after diagnosis as compared to patients with PM from left‐sided tumors. In patients treated with palliative chemotherapy right‐sided primary tumor was associated with impaired survival. This suggests that treatment with chemotherapy is less effective in patients with right‐sided primary tumors, which is concordant with previously published studies which included mainly patients without PM.^20^, ^21^, ^34^ Median OS of palliative patients not treated with chemotherapy was dismal (<3 months). In these patients, there was no significant difference between left‐ and right‐sided tumors.

The baseline characteristics of this study population are comparable with those previously reported on patients with metastatic colon cancer. Prior research also showed that right‐sided primary tumors are more frequently found in females, diagnosed at a more advanced stage and are more likely to be characterized by mucinous histology.^13^, ^15^, ^22^, ^35^, ^36^ Other prognostic factors observed were in line with the literature as well.

Using data retrieved from the NCR has many advantages. These include the sample size, the population‐based design and, as a consequence, the ability to report on all patients with synchronous PM, as opposed to many studies that focus on a specific treatments such as CRS‐HIPEC. But, inherently to the design and the data source this study has limitations. The NCR only collected data on patients with synchronous PM of colon cancer. Therefore, no conclusions can be drawn for patients with metachronous PM. The NCR also did not collect data on the extent of peritoneal disease scored with the peritoneal cancer index (PCI) or on the completeness of cytoreduction during CRS‐HIPEC, which are both well‐known prognostic factors after CRS‐HIPEC. Mutational status and microsatellite status of the tumor was not widely available. While prior research has shown that these are all prognostic factors for patients with metastatic colon cancer, and right‐sided tumors have been associated with more BRAF/RAS mutations.^34^, ^37^, ^38^, ^39^, ^40^, ^41^ Consequently, the results of this study should be interpreted with care.

In conclusion, this population‐based study showed that patients with synchronous PM originating from right‐sided colon cancer have an impaired OS as compared to left‐sided primary tumors. However, after CRS‐HIPEC there is no significant difference in the outcomes of right‐ and left‐sided primary tumors.

## CONFLICT OF INTEREST

The authors declare no conflicts of interest.

## AUTHORS’ CONTRIBUTIONS

JWAB, IHJTdH, and CV initiated this study. NLdeB, KR, and AB wrote the research proposal and submitted the application to the NCR. NLdB and AB performed the statistical analysis. NLdB and KR took the lead in drafting this manuscript. JWAB, EVEM, ARMB, NFMK, JHWTdW, PHdR, and IHJTdH are experienced CRS‐HIPEC surgeons in the participating hospitals. All authors, NLdB, KR, JWAB, EVEM, ARMB, NFMK, JHWTdW, PHdR, AB, IHJTdH, and CV revised and contributed to the manuscript. All authors approved the final version of the manuscript. CV supervised the project.

## Supporting information

Fig S1A

Fig S1B

Fig S1C

Table S1‐S2

## Data Availability

The data that support the findings of this study are available from the NCR. Restrictions apply to the availability of these data, which were used under license for this study. Data are available only with the permission of the NCR Monitoring Committee.
