# Supplementary figures and images for: A population‐based study on the prognostic impact of primary tumor sidedness in patients with peritoneal metastases from colon cancer
Source: Cancer Med. 2020 Jul 2;9(16):5851–9. doi: 10.1002/cam4.3243 (PMC7433839; doi:10.1002/cam4.3243)

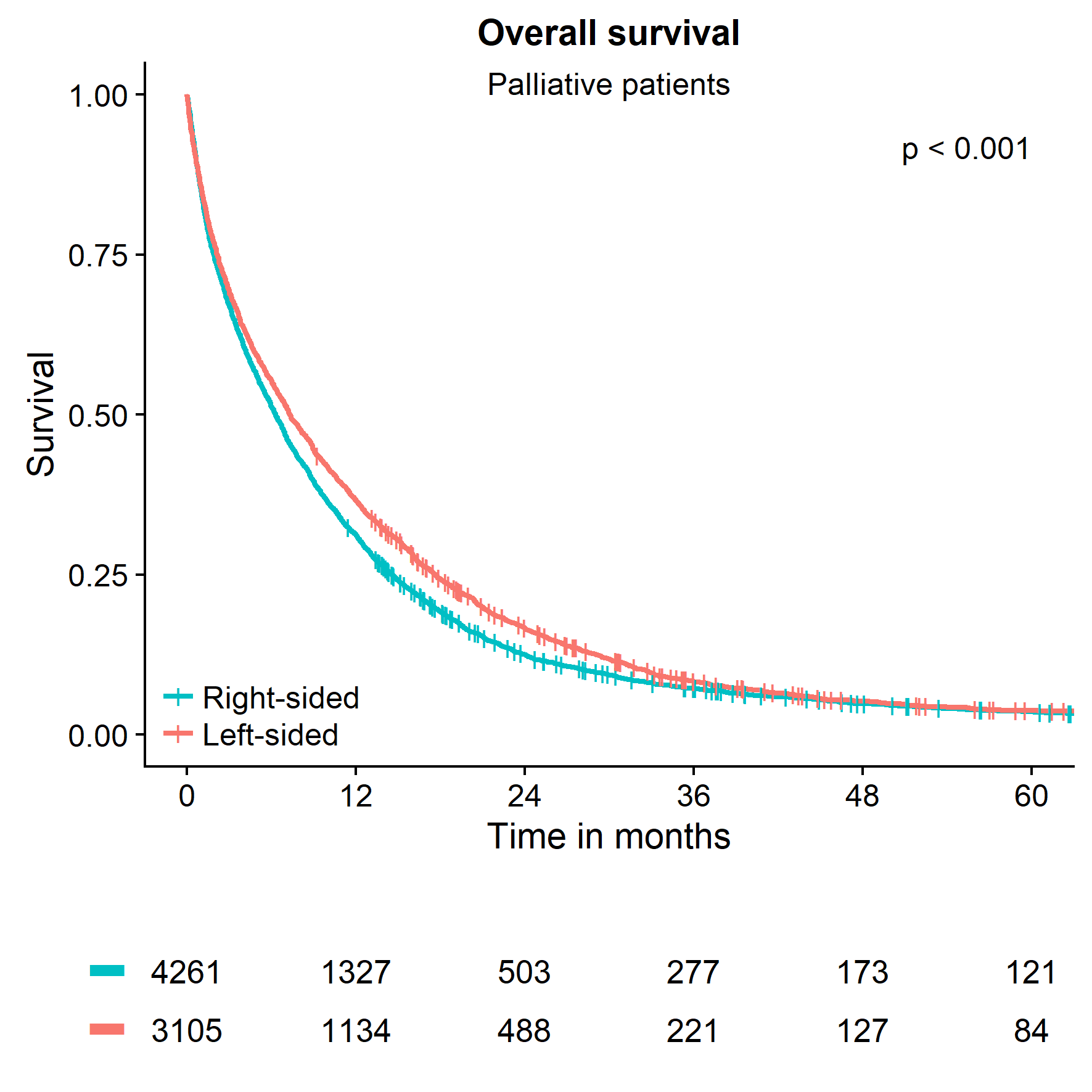

Supplement: Supplementary file 1 — Fig S1A [file CAM4-9-5851-s001.png]

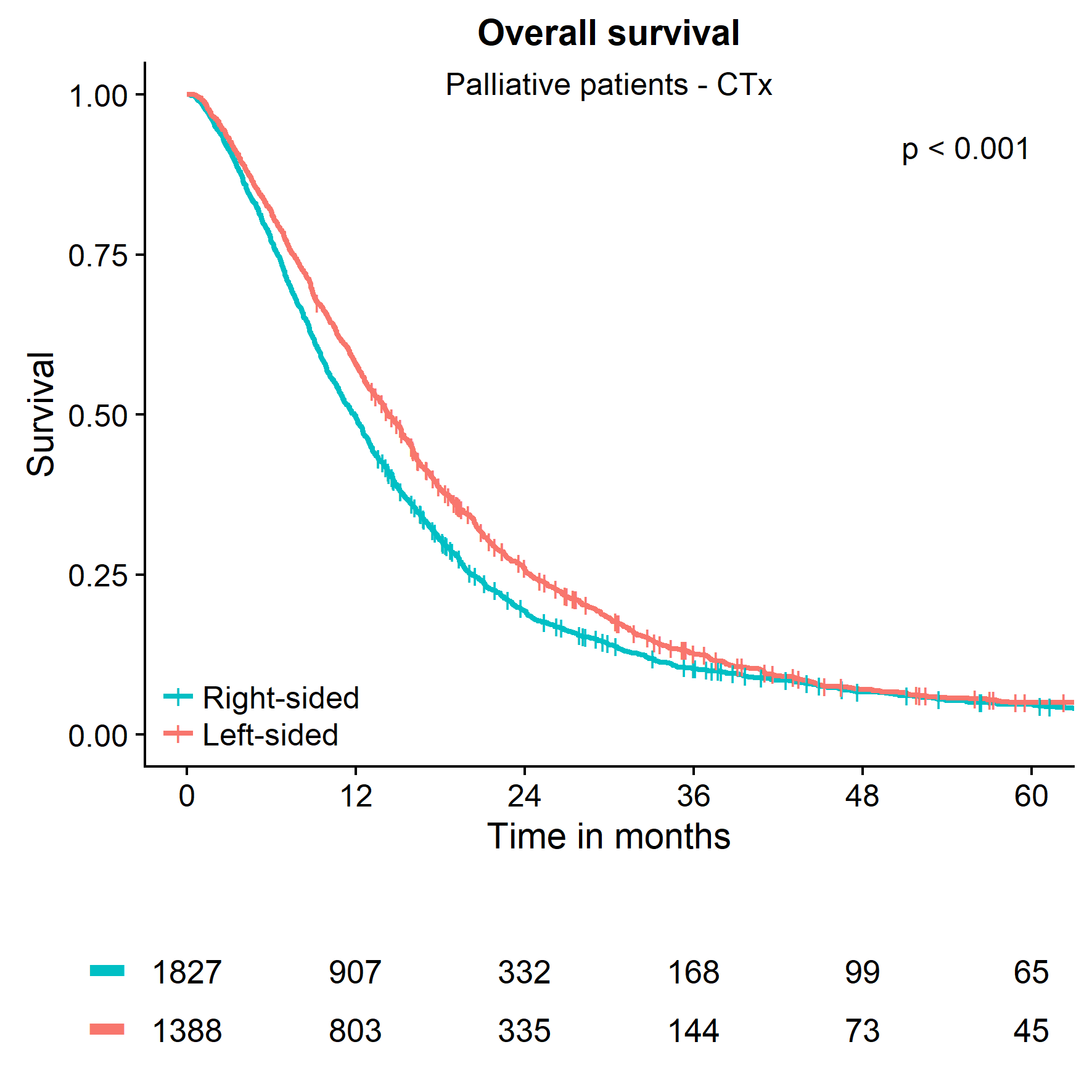

Supplement: Supplementary file 2 — Fig S1B [file CAM4-9-5851-s002.png]

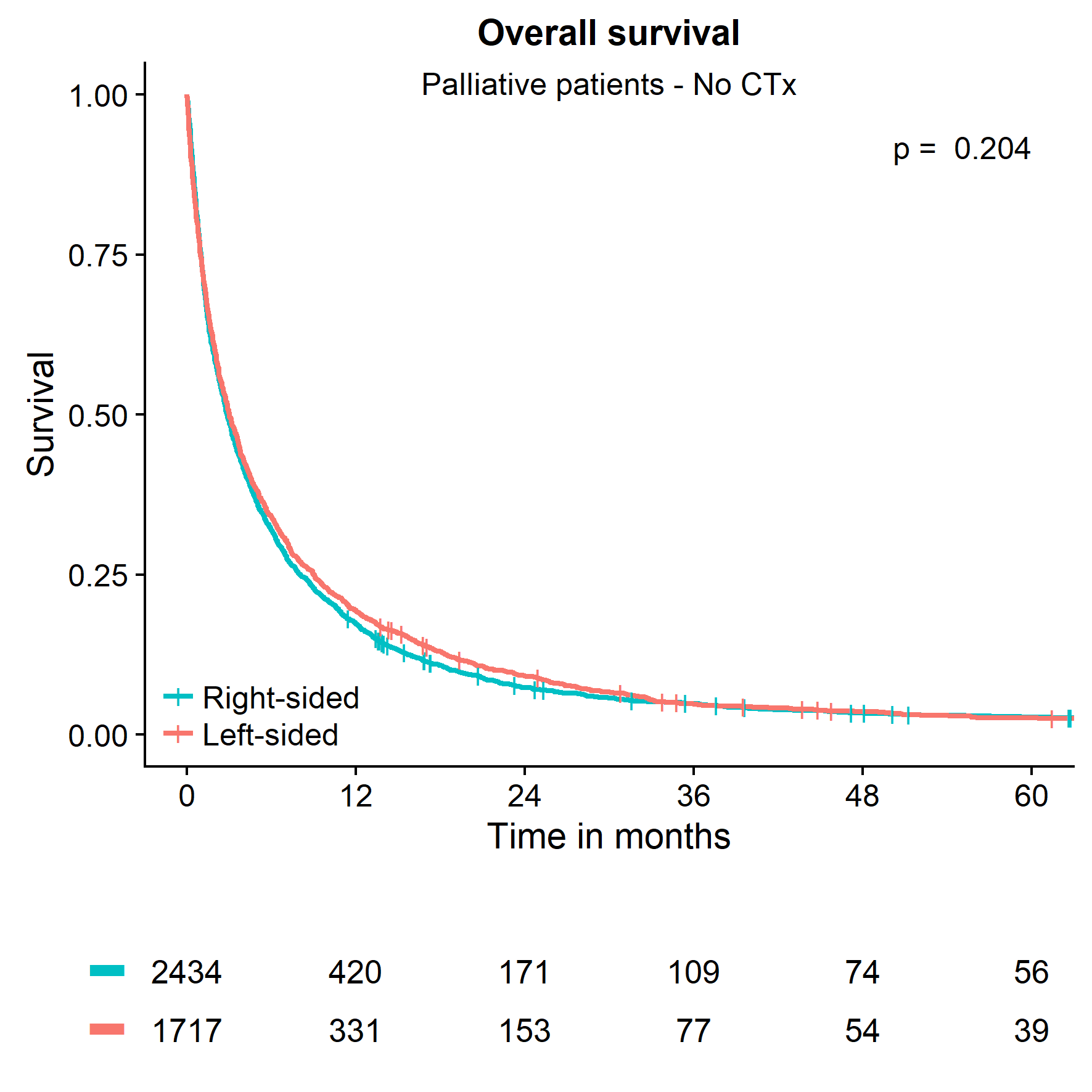

Supplement: Supplementary file 3 — Fig S1C [file CAM4-9-5851-s003.png]
