# Supplementary material for: A population‐based study on the prognostic impact of primary tumor sidedness in patients with peritoneal metastases from colon cancer
Source: Cancer Med. 2020 Jul 2;9(16):5851–9. doi: 10.1002/cam4.3243 (PMC7433839; doi:10.1002/cam4.3243)
Supplement: Supplementary file 4 — Table S1‐S2 [file CAM4-9-5851-s004.docx]

**Supplementary table 1.** Baseline characteristics of patients receiving palliative treatment

| *Baseline variables* | **Right-sided**  N= 4261 (57.8%)  Median [IQR] /N (%) | **Left-sided**  N= 3105 (42.2%)  Median[IQR]/N (%) | *p* value |
| --- | --- | --- | --- |
| **Age at diagnosis** | 71 [63-78] | 69 [61-78] | **<0.001** |
| **Sex** |  |  |  |
| Male | 1979 (46.4) | 1687 (54.3) | **<0.001** |
| Female | 2282 (53.6) | 1418 (45.7) |  |
| **Year of incidence** |  |  | **0.014** |
| 1995 – 1999 | 410 (55.9) | 323 (44.1) |  |
| 2000 – 2004 | 529 (54.0) | 451 (46.0) |  |
| 2005 – 2009 | 1246 (57.7) | 913 (42.3) |  |
| ≥2010 (2016) | 2076 (59.4) | 1418 (40.6) |  |
| **Location primary** |  |  |  |
| Cecum | 2006 (47.1) | - |  |
| Ascending colon | 1118 (26.2) | - |  |
| Hepatic flexure | 469 (11.0) | - |  |
| Transverse colon | 668 (15.7) | - |  |
| Splenic flexure | - | 355 (11.4) |  |
| Descending colon | - | 326 (10.5) |  |
| Sigmoid colon | - | 2096 (67.5) |  |
| Recto sigmoid | - | 328 (10.6) |  |
| **pT-stage primary** |  |  | 0.070 |
| T1-2 | 25 (0.6) | 28(0.9) |  |
| T3-4 | 2085 (48.9) | 1420 (45.7) |  |
| *Unknown* | *2151 (50.5)* | *1657 (53.4)* |  |
| **pN-stage primary** |  |  |  |
| N0 | 222 (5.2) | 217 (7.0) | **<0.001** |
| N+ | 1799 (42.2) | 1145 (36.9) |  |
| *Unknown* | *2240 (52.6)* | *1743 (56.1)* |  |
| **Extra-peritoneal metastasis** |  |  | **<0.001** |
| Yes | 2094 (49.1) | 1695 (54.6) |  |
| No | 2167 (50.9) | 1410 (45.4) |  |
| **Mucinous histology** |  |  | 0.811 |
| Yes | 818 (19.2) | 603 (19.4) |  |
| No | 3444 (80.8) | 2502 (80.6) |  |
| **Differentiation** |  |  | **<0.001** |
| Well | 104 (2.4) | 100 (3.2) |  |
| Moderate | 1309 (30.7) | 1122 (36.1) |  |
| Poor | 1231(28.5) | 677 (21.8) |  |
| *Unknown* | *1635 (38.4)* | *1206 (38.8)* |  |
| **CTx** |  |  | 0.119 |
| Yes | 1827(42.9) | 1388 (44.7) |  |
| No | 2434 (57.1) | 1717 (55.3) |  |
| **Surgery of primary** |  |  | **0.024** |
| Yes | 2125(50.0) | 1467 (47.3) |  |
| No | 2128(50.0) | 1635 (52.7) |  |

**CTx* chemotherapy, *CRS-HIPEC* cytoreductive surgery and hyperthermic intraperitoneal chemoterapy

**Supplementary table 2.** Univariable and Multivariable Cox regression analysis for OS patients receiving palliative treatment

| Factor | Univariable analysis | | | Multivariable analysis | | |
| --- | --- | --- | --- | --- | --- | --- |
|  | HR | 95% CI | *p* value | HR | 95% CI | *p* value |
| Age at diagnosis (cont.) | 1.02 | 1.02 - 1.02 | **<0.001** | 1.011 | 1.01 - 1.02 | **<0.001** |
| Female sex | 1.07 | 1.02 - 1.12 | **0.004** | 1.025 | 0.95 - 1.11 | 0.521 |
| Year of incidence ≥2010 | 0.98 | 0.94 - 1.03 | 0.417 |  |  |  |
| Right-sided primary | 1.11 | 1.06 - 1.16 | **<0.001** | 1.09 | 1.01 - 1.18 | **0.033** |
| pT3-T4 (primary) | 1.87 | 1.39 - 2.50 | **<0.001** | 2.10 | 1.44 - 3.06 | **<0.001** |
| pN+ CRC | 1.66 | 1.49 - 1.85 | **<0.001** | 1.83 | 1.62 - 2.06 | **<0.001** |
| Extra-peritoneal metastasis | 1.23 | 1.17 - 1.29 | **<0.001** | 1.83 | 1.68 - 1.98 | **<0.001** |
| Poor differentiation | 1.47 | 1.38 - 1.56 | **<0.001** | 1.44 | 1.33 - 1.55 | **<0.001** |
| Mucinous histology | 0.86 | 0.81 - 0.91 | **<0.001** | 1.04 | 0.94 - 1.14 | 0.437 |
| Chemotherapy | 0.46 | 0.44 - 0.49 | **<0.001** | 0.45 | 0.41 - 0.49 | **<0.001** |
| Surgery | 0.45 | 0.43 - 0.47 | **<0.001** | 0.79 | 0.29 - 2.11 | 0.633 |

** HR* hazard ratio, *CI* confidence interval, *pN+* lymph node positives, *CRC* colorectal cancer

**Supplementary figure 1.** Overall survival of patients with synchronous peritoneal metastases from colon cancer receiving palliative treatment a) Overal survival of all patients receiving palliative treatment. b) Overall survival of patients treated with palliative systemic chemotherapy. c) Overall survival of palliative patients not treated with systemic chemotherapy.
**CTx* Chemotherapy
